# Microvascular Resistance Reserve (MRR): a new concept to understand the coronary microcirculation

**DOI:** 10.1007/s12471-026-02026-w

**Published:** 2026-02-26

**Authors:** Annemiek de Vos, Danielle Keulards, Tijn Jansen, Caïa Crooijmans, Peter Damman, Pim Tonino

**Affiliations:** 1 Department of Cardiology, Catharina Hospital, Eindhoven, The Netherlands; 2 Department of Cardiology, Radboud University Medical Centre, Nijmegen, The Netherlands

**Keywords:** ANOCA, Coronary physiology, Absolute coronary blood flow, Coronary function test, Microcirculatory resistance, Microcirculatory resistance reserve

## Abstract

In recent years, there have been numerous developments in the field of coronary physiology with the addition of new indices for the evaluation of the coronary microcirculation, such as invasive measurement of absolute coronary blood flow and microvascular resistance. This has resulted in more accurate diagnostic tools for patients with angina and no obstructive coronary artery disease (ANOCA), with the possibility to distinguish different endotypes of coronary microvascular dysfunction (CMD). Because of the growing recognition of ANOCA and, along with that, the increasing application of coronary function testing due to the latest guideline recommendations, it is timely and important to further explain the concept of absolute coronary flow, microvascular resistance, and the latest addition to the field: Microvascular Resistance Reserve (MRR). Differentiation between specific endotypes of CMD will help to develop tailored therapy for ANOCA patients.

## Introduction

In the 2024 ESC guideline for the management of chronic coronary syndromes, an expanded section is dedicated to the diagnosis and treatment of patients with angina and no obstructive coronary artery disease (ANOCA) [1]. This is completely befitting the evolving and growing insights into the prevalence and importance of this clinical entity, and the developments that have been made in understanding the underlying mechanisms and different endotypes in the past years [2]. In the current guidelines, there is now a Class IB recommendation to perform complete coronary function testing (CFT) in suspected ANOCA patients who are persistently symptomatic despite medical treatment. Treatment options for ANOCA patients are still limited, whether suffering from vasospastic angina, microvascular dysfunction (CMD), or a combination of both. For further developments in tailored therapy and understanding of the complex mechanisms that are at play in the coronary microcirculation, it is paramount that the indices used for diagnosing these different endotypes are as specific and accurate as possible.

In the current protocol for complete coronary function testing, coronary flow reserve (CFR) and the index of microvascular resistance (IMR), are the standard indices for invasive assessment of microvascular function. CFR represents the reserve capacity of the coronary circulation (meaning: what is the capacity of the vessel to increase blood flow from resting state to maximal/hyperemic state?), and can be measured invasively with bolus and continuous thermodilution techniques. CFR encompasses coronary blood flow throughout all segments of the coronary circulation and is therefore not specific for the microcirculation. IMR represents the minimal attainable resistance of the microcirculation and is calculated by multiplying distal coronary pressure by the mean transit time during hyperemia. When Doppler velocity sensor wires are used, one can also measure Coronary Flow Velocity Reserve (CFVR) and the hyperemic microvascular resistance index (HMR), being the ‘Doppler-based counterparts’ of CFR and IMR. Details regarding CFVR and HMR are beyond the scope of this manuscript [3].[4]. IMR has shown to be a good predictor of prognosis, and although it is rather specific for the microcirculation, it needs correction with a higher degree of coronary stenosis. The technical difficulty—even for experienced operators—of obtaining high-quality and reproducible measurements with bolus thermodilution and Doppler drove further developments in measuring flow and resistance [5, 6]. The need for adenosine often causes discomfort for patients during the measurements and can be a problem in patients with severe asthma. Other (non-invasive) indices, such as angiography-derived IMR (angio-IMR, which has shown to have a good correlation with wire-based IMR [7, 8]) look promising in terms of simplifying the procedure, but will still have the limitation of being a surrogate parameter for flow and/or resistance.

This has led to the development of the continuous thermodilution method, which enables direct quantification of true (absolute) coronary blood flow (Q, ml/min) and microvascular resistance (Rµ, Wood Units or mm Hg/L/min). Recently, it has become possible to measure Q and Rµ not only at hyperemia but also at rest [9]. Subsequently, absolute CFR and so-called Microvascular Resistance Reserve (MRR) have become available. CFR is the classical ratio of maximum blood flow and resting blood flow (and as such, not specific to the microcirculation and confounded by epicardial disease), whereas MRR is the ratio of true resting microvascular resistance (not confounded by epicardial disease) and hyperemic microvascular resistance [10]. MRR can be calculated with all sorts of modalities involving distal and proximal coronary flow and pressure measurements at rest and in a hyperemic state, and is independent of myocardial mass.

Extensive research during the last decade has already shown that measuring absolute flow and resistance by continuous thermodilution has a better association with symptoms and higher reproducibility than bolus thermodilution and Doppler [11–15]. With the addition of MRR as the most specific index of microvascular dysfunction, another step is taken towards better evaluation and treatment of ANOCA patients. Because of the growing recognition of ANOCA and, along with that, the increasing application of CFT due to the latest guideline recommendations, we feel it is timely to further explain the concept of absolute coronary flow, microvascular resistance, and MRR to a wide cardiology audience. It is also important to emphasize that obtaining such measurements in the cathlab is easy to learn, safe, and that it is quite simple to implement continuous thermodilution and MRR for those already performing CFT with bolus thermodilution techniques.

## The principles of measuring flow with continuous thermodilution

**Fig. 1:**
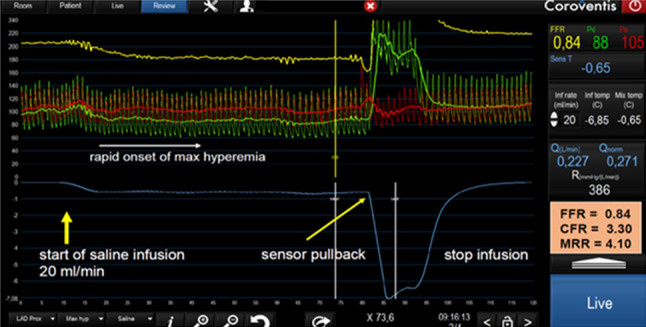
Example of continuous thermodilution in hyperemic conditions

The first practical steps for measuring flow and microvascular resistance are identical to performing FFR. After advancing a guiding catheter and, after equalization, a standard pressure-temperature wire (Pressure wire X, Abbott, Minneapolis) is placed in the distal part of the coronary artery. Next, a specifically designed 2.2 Fr multisidehole monorail infusion catheter (Rayflow, Hexacath, Paris) is advanced over the pressure wire and located with its tip at the desired place in the coronary artery. The position of the infusion catheter determines the area of myocardial mass that is investigated. In routine CFT in ANOCA patients, the infusion catheter is positioned in the proximal LAD. Through the infusion catheter, saline is infused by an automatic high pressure pump (not to be confused with a regular infusion pump as is used for intravenous use, but a pump that can generate pressures up to 350 psi) at a predetermined infusion rate, being 10 ml/min for measurements in resting conditions (Q_*rest*_ and R_*µ,rest*_), and 20 ml/min for hyperemic conditions (Q_*hyper*_ and R_*µ,hyper*_) [16]. After complete mixing of the saline with blood, distal coronary temperature is measured (T), and as soon as a steady state for T is achieved, the temperature sensor is pulled back to the tip of the infusion catheter, where the temperature of the saline (Ti) is measured when it enters the coronary artery (example in Fig. 1). Absolute coronary blood flow is then calculated by the equation $Q=Qi \left(\frac{Ti }{T}\right)\cdot 1.08$. The correction factor 1.08 accounts for the difference in specific heat between blood and saline. And microvascular resistance R_*µ*_ is calculated as P_*d*_/Q (in mm Hg/l/min or WU). The use of a dedicated infusion catheter is important to ensure complete and homogenous mixing of blood and saline throughout the artery, which is essential for accurate measurements [17]. When the temperature and rate of the infused saline are known, as well as the temperature of the mixture, it is possible to calculate the blood flow and microvascular resistance. All calculations are automatically performed by dedicated software (CoroFlow, Coroventis). Further practical details and performance of the procedure have been described in great detail by Belmonte et al. [14]. Although in standard coronary function testing all measurements are done in the LAD, it is also possible to measure absolute flow and resistance in the RCA and RCX. Depending on the caliber and perfusion territory of the artery, infusion rates can be set to 15 ml/min for the RCA and 8 ml/min for the RCX. In large RCA or RCX, it is also safe to start at 20 and 10 ml/min.

Red and green lines represent aortic pressure and distal coronary pressure as usual. The blue line represents distal coronary temperature, measured by the pressure-temperature sensor on the wire. On the very left-hand side of the tracing, the resting situation is displayed. To obtain hyperemic flow and resistance, the infusion rate of saline is set to 20 ml/min, creating maximum hyperemia. In this example, FFR equals 0.84, maximum flow equals 227 ml/min and microvascular resistance of the complete anterior wall is 386 WU (this is within the normal range for LAD). It is important to note that no separate hyperemic stimulus is mandatory because intracoronary saline at a rate of 20 ml/min creates maximum hyperemia. When resting measurements are performed as well by setting the infusion rate of saline to 10 ml/min, it is possible to calculate CFR and MRR (being 3.3 and 4.1 in this case).

## Microvascular Resistance Reserve (MRR)

**Fig. 2:**
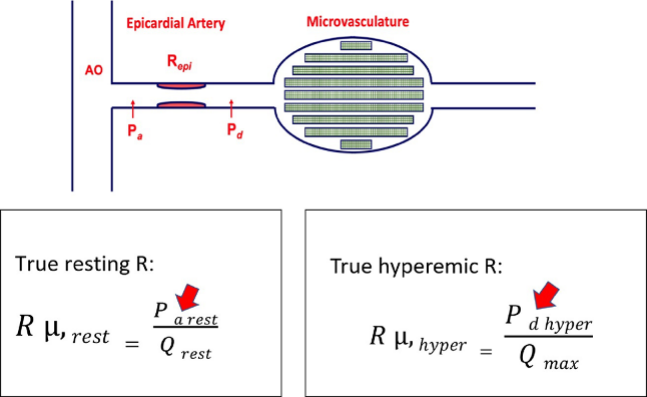
Schematic display of a coronary artery with its adjoining microvasculature. Following Ohm’s law, in the presence of epicardial disease, the measured resting microvascular resistance equals P_*d*_/Q_*rest*_, but it is important to realize that true resting microvascular resistance is given by P_*a*_/Q_*rest*_. Hyperemic microvascular resistance equals P_*d*_ at hyperemia/Q_*max*_ (irrespective of epicardial disease)

**Fig. 3:**
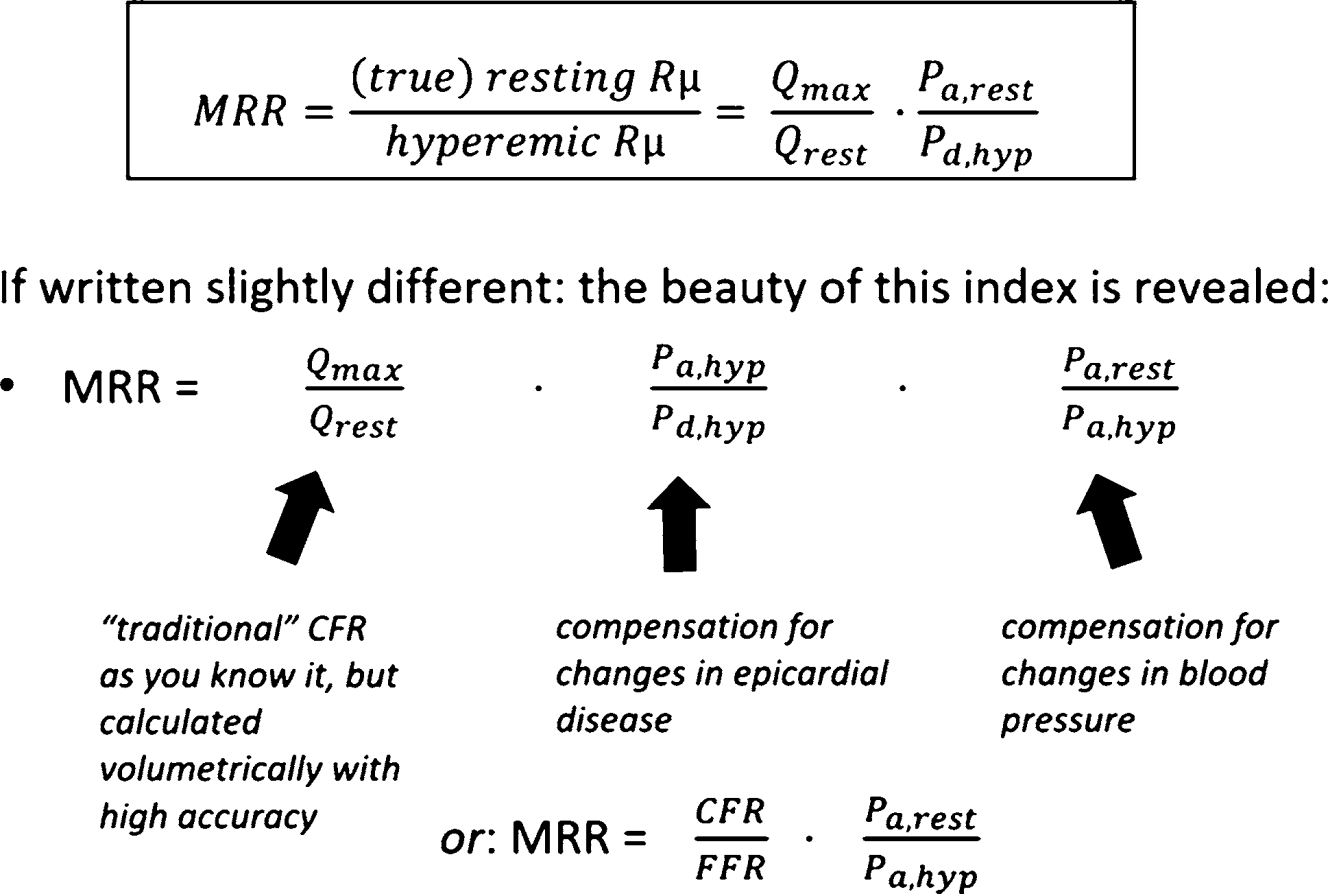
Upper formula: MRR equals the ratio of true resting and hyperemic resistance, as defined in Fig. 2, being the ratio of hyperemic and resting blood flow, multiplied by proximal pressure (P_*a*_) at rest and divided by distal pressure (P_*d*_) at hyperemia. By multiplying both the numerator and denominator by the same term P_*a,hyp*_, the equation for MRR becomes more intuitively clear. The first term in this MRR-equation is classical CFR (Q_*max*_/Q_*rest*_), the second term is the inverse value of FFR (P_*a,hyper*_/P_*d,hyper*_) and compensates for the presence of epicardial disease. The third term is a compensation for changes in blood pressure between resting and hyperemic measurement (P_*a,rest*_/P_*a,hyper*_). Consequently, this equation can also be written as: MRR = (CFR/FFR) × (P_*a, rest*_/P_*a, hyper*_), which is a universal way to express the status of the microcirculation. If P_*a,rest*_ is equal to P_*a,hyper*_, as is generally the case during saline-induced hyperemia, this last equation can be further simplified to: MRR = CFR/FFR. If hyperemia is induced by adenosine or other drugs, P_*a,rest,*_ and P_*a,hyper*_ are usually different by 10–30%, and the second term of the equation needs to be taken into account to assess the mutual relation between MRR, FFR, and CFR

From the absolute flow measurements, FFR, and simultaneously measured pressures, the index Microvascular Resistance Reserve (MRR) can be calculated, which is an accurate, specific, and mass-independent index of the coronary microcirculation. It is defined as the ratio of maximum blood flow to true resting blood flow, as it would be in the hypothetical case that the epicardial artery is completely normal. The concept of MRR was introduced by Pijls in 2021, and the theoretical framework of calculating true resting R_*µ*_, hyperaemic R_*µ*_, MRR, and the relation among MRR, FFR, and CFR has been described in detail by de Bruyne, Pijls, Belmonte, and Keulards [10, 14]. Even with the current possibility to measure coronary blood flow Q and microvascular resistance R_µ_ accurately, it should be realized that the actually measured resting R_*µ*_ in any myocardial territory is influenced by the presence of epicardial disease. Such epicardial disease—whether focal or diffuse, mild or severe, or induced by the presence of a catheter or guidewire—will lead to compensatory microvascular dilatation as a result of coronary autoregulation. This physiological phenomenon means that for any given increase in epicardial resistance, an equivalent decrease of resting microvascular resistance will occur to maintain resting flow to the myocardium constant. Consequently, the *actually measured* resting resistance is not a true reflection of resting resistance, and therefore, a simple ratio between hyperemic and actually measured resting resistance is not sufficient to characterize the microcirculation. What one needs to truly understand the microcirculation is an index that represents the relation between hyperemic microvascular resistance and the resting microvascular resistance, *as it would be in the hypothetical case that the epicardial circulation were completely normal. *This was labelled ‘*true resting R*_*µ*_*’* and from the theoretical background as explained by De Bruyne and Pijls in the seminal paper on MRR [10], it becomes clear that MRR assesses the microcirculation *specifically*, independent of epicardial disease, hemodynamic variations, and myocardial mass. This physiological framework is visualised and explained in Figs. 2 and 3.

## Considerations on Microvascular Resistance Reserve and some limitations

In the sections above, we already outlined why MRR is the first and so far, only index that is specific for the microcirculation and independent of epicardial stenosis. It is also more accurate and reproducible than bolus-derived IMR and CFR, and continuous thermodilution has proven to be safe [18]. On top of that, when both resting flow and resistance are measured and can be compared to hyperemic flow and resistance, it becomes possible to distinguish between functional and structural abnormalities of the microcirculation in a patient. Functional CMD due to endothelial dysfunction with impaired vasodilatory response results in elevated resting blood flow (Q) and decreased microvascular resistance (R_*µ*_) at rest, but normal values at hyperemia. Structural CMD, on the other hand, is reflected by decreased hyperemic blood flow (Q) and increased hyperemic resistance (R_*µ*_) [19]. This subdivision is important for understanding and diagnosing pathological disorders of the microcirculation, but especially for future research and development of better therapies for ANOCA patients. We have come a long way since ANOCA was poorly understood, referred to as ‘Syndrome X’ or over 20 different definitions, resulting in a very heterogeneous group of patients, which made it nearly impossible to develop treatment or good clinical guidelines for all of these patients. With the present armamentarium of physiological indices, this has changed dramatically. Although at this point it should be emphasized that MRR can theoretically also be calculated by using bolus thermodilution techniques, the strength of any device is not stronger than its weakest link. MRR calculated in such a way is, by definition, suboptimal compared to MRR calculated by absolute flow measurements. In addition, without absolute flow measurements, no quantitative value of Q (in ml/min) and R_*µ*_ (in WU) are available, and the distinction between functional and structural microvascular disease remains difficult. Therefore, using absolute flow measurements and continuous thermodilution remains preferable for optimal assessment of the coronary microcirculation.

For the implementation of absolute flow and resistance measurements and MRR in the cathlab some specific equipments are required. For cathlabs already performing bolus thermodilution in CFT, most materials will be present already, such as the pressure wire, high-pressure infusion pump, and Coroventis software. However, the dedicated Rayflow ®infusion catheter (Hexacath, Paris) is mandatory for a complete and homogenous mixture in the investigated artery. It should also be stressed that a high-pressure infusion pump should be used for saline infusion, like a contrast injector is used in left ventricular angiography. Such a pump should be programmed in the infusion mode (ml/min) and not in its injector mode (ml/sec). Although the technique is very easy to learn, some centres will find the cost of the necessary equipment, as well as being unfamiliar with the measurements, an impediment to starting to measure absolute coronary blood flow and microvascular resistance by continuous thermodilution. It is understandable that any new index needs time before growing from pioneering to becoming a tool being fully adopted in daily practice, as was the case with FFR. There is now a small but expanding body of evidence showing the accuracy and added value of MRR, and this will lead to cardiologists becoming more and more accustomed to these parameters.

Outside of the cathlab, MRR can be approximated as well. The equation showing that MRR = CFR/FFR × P_*a,rest*_/P_*a,hyp*_, illustrates that non-invasively obtained measurements of CFR and FFR by CT and PET can also be used to calculate MRR, if at least P_*a*_ at rest and hyperemia are carefully measured non-invasively during PET. Further developments in measuring MRR by combined parameters, for instance, CT-derived FFR and CFR from PET-CT are being investigated. One should realise, however, that a complete Coronary Function Test also contains Acetylcholine-based testing for epicardial coronary spasm. Unfortunately, that diagnostic part of CFT is difficult to acquire in a non-invasive manner.

Indices with one true cut-off point between ‘health and disease’ are rare in clinical medicine. For example, FFR equals ∿ 1.0 in every normal patient and every normal coronary artery. Therefore, a sharp cut-off value for FFR can be expected (around 0.8) with minimal grey zone. MRR shares the disadvantage with CFR to be age-dependent and shows some heterogeneity. Therefore, by definition, a grey zone exists between strictly normal and abnormal values. Presently available data suggest that with MRR-values above 3.0, microvascular disease can be excluded and that below a value of 2.3, microvascular disease is likely to be present [20, 21]. We expect further research to identify the correct boundaries for MRR in diagnosing microvascular dysfunction.

## Conclusion

For suspected ANOCA patients, correctly diagnosing or ruling out microvascular dysfunction is important. Measuring absolute coronary blood flow and microvascular resistance, with the additional calculation of Microvascular Resistance Reserve (MRR), is the most accurate and specific method for that purpose that is now available. With the new guidelines and recommendations for performing complete coronary function testing in suspected ANOCA patients, it is important for cardiologists to have knowledge of the different available indices and their characteristics. For operators already performing CFT with bolus thermodilution in the cathlab, it is easy and feasible to implement continuous thermodilution and MRR. Differentiation between specific endotypes of CMD will help to develop tailored therapy for ANOCA patients.
